# A Therapeutic Approach to Nasopharyngeal Carcinomas by DNAzymes Targeting EBV LMP-1 Gene

**DOI:** 10.3390/molecules15096127

**Published:** 2010-09-01

**Authors:** Lifang Yang, Zhongxin Lu, Xiaoqian Ma, Ya Cao, Lun-Quan Sun

**Affiliations:** 1 Cancer Research Institute, Xiangya School of Medicine, Central South University, Changsha, Hunan 410078, China; E-Mail: yanglifang99@hotmail (L.Y.); 2 Center for Molecular Medicine, Xiangya Hospital, Central South University, Changsha, Hunan 410078, China; 3 Department of Medical and Molecular Biosciences, University of Technology Sydney, Australia

**Keywords:** LMP1, DNAzyme, NPC, targeted therapy

## Abstract

Epstein-Barr virus (EBV)-encoded latent membrane protein 1 (LMP1) has been known to have oncogenic properties during latent infection in nasopharyngeal carcinoma (NPC). Genetic manipulation of LMP1 expression may provide a novel strategy for the treatment of NPC. DNAzymes are synthetic, single-stranded DNA catalysts that can be engineered to bind and cleave the target mRNA of a disease-causing gene. By targeting the LMP1 mRNA, we successfully obtained a phosphorothioate-modified ‘‘10–23’’ DNAzyme namely DZ1, through screening a series of DNAzymes. DZ1 could significantly down-regulate the expression of LMP1 in NPC cells, inhibit cell proliferation, metastasis, promote apoptosis and enhance radiosensitivity of NPC through interfering signal pathways which are abnormally activated by LMP1, including NF-κB, AP-1 and STAT3 signal pathways. Together, interfering LMP1 signaling pathway could be a promising strategy to target the malignant phenotypes of NPC.

## 1. Introduction

Nasopharyngeal carcinoma (NPC), a malignancy arising from the epithelium lining of the posterior nasopharynx, is endemic in Southern China and Southeast Asia, with a striking racial and geographic distribution and has caused very serious health problem in these areas [1]. Epstein-Barr virus (EBV) is a lymphotropic human gamma herpesvirus which infects more than 90% individuals in the human population and has been implicated in the pathogenesis of several human malignancies including Burkitt’s and Hodgkin’s lymphomas, gastric carcinoma and NPC. EBV infection in NPC is classified as type II latent infection in which only EBV nuclear antigen-1(EBNA-1), latent membrane protein-1(LMP1), LMP2, and EBV early RNA (EBER) expressions can be detected [2,3]. Among these proteins, LMP1 is thought to play a key role in the pathogenesis of NPC [4,5]. LMP1 is an integral membrane protein composed of a short intracellular N terminus, six hydrophobic transmembrane domains, and an intracellular carboxyl-terminal activating region (CTAR) including three functional domains, CTAR1, CTAR2, and CTAR3. LMP1 associates with tumor necrosis factor receptor-associated factors (TRAFs), tumor necrosis factor receptor-associated death domain protein (TRADD), and receptor-interacting protein (RIP). LMP1 activates different signal transduction pathways that include nuclear factor kappa B (NF-κB), c-jun N-terminal kinases (JNK)/c-Jun/activator protein 1 (AP-1), mitogen-activated protein kinases (p38-MAPK)/activating transcriptional factor (ATF), and Janus kinase (JAK)/ signal transducers and activators of transcription protein (STAT) [6,7,8], and causes various downstream pathological changes in cell proliferation, anti-apoptosis and metastasis [7,9,10,11]. Therefore, genetic manipulation of the LMP1 expression may provide a novel strategy for the treatment against NPC. 

An ideal oligonucleotide based gene inactivation agent targeting RNA would combine the self sufficient RNA digestion capability of ribozymes such as the “hammerhead” and the “hairpin”, with the relative biological resilience of the antisense oligodeoxynucleotides (ODN). Although DNA molecules with RNA cleavage activity have not been observed in nature, some have been derived as a result of an artificial evolutionary system known as *in vitro* selection [12,13,14,15]. In an *in vitro* selection system, DNA liberated from its complementary strand is free to explore a full range of structural possibilities, some of which have been found to be capable of catalytic activity, including site specific RNA cleavage and ligation [16,17].

The 10-23 DNA enzyme or DNAzyme was named from its origin as the 23rd clone characterised from the 10th cycle of *in vitro* selection [14]. This enzyme has a number of features, which endow it with tremendous potential for applications both *in vitro* and *in vivo*. These include its ability to cleave almost any RNA sequence with high specificity provided it contains a purine-pyrimidine dinucleotide. This can be accomplished at very high kinetic efficiency with rates approaching and even exceeding those of other nucleic acid and protein endoribonucleases [14]. The ability of the 10-23 DNAzyme to specifically cleave RNA with high efficiency under simulated physiological conditions has fuelled expectation that this agent may have useful biological application in a gene inactivation strategy [18,19]. To explore this potential a number of groups have attempted to examine the activity of DNAzymes in biological systems [20,21,22]. In this review, we canvass the use of catalytic DNA in the inhibition of EBV LMP1 gene expression and provide a summary of our research in the field, followed by a discussion of the potential for the use of DNAzymes for therapeutic approaches to NPC.

## 2. Results and Discussion

### 2.1. Design and Chemical Modifications of LMP1 Targeted DNAzymes

Like other agents that function by hybridization with single-stranded RNA, the DNAzyme must compete with the target’s own stable intramolecular base pairing, which forms its characteristic secondary structures. Fortunately, the 10-23 DNAzyme cleavage sites are plentiful in most biological substrates and thus provide a host of opportunities to achieve maximum cleavage efficiency. EBV has been shown to possess some mutations in the LMP1 gene and these sequence changes may be associated with the viral oncogenic potential. In order to identify some conserved regions in the LMP1 gene for DNAzyme targeting, we aligned the LMP1 sequences with a panel of eight EBV isolates, including representatives of four defined groups of European LMP1 variants (groups A-D) and Chinese NPC-derived LMP1 [23]. In addition to target site accessibility, the thermodynamics of enzyme-substrate interactions also have a strong influence on the efficiency of DNAzyme mediated hydrolysis of a long RNA [24]. Taking into consideration these factors, we designed 13 different DNAzymes targeted against AU or GU sites along the full-length LMP1 mRNA. All DNAzymes contained the 10–23 catalytic motif (ggctagctacaacg) and 9 bp hybridizing arms specific for RU cleavage sites (R=G or A) in the conserved regions of the LMP1 transcript. DNAzymes were selected if their hybridization stability with their targets (free energy of hybridization, ΔG^0^) is <-25 kcal/mol [25]. DNAzymes were synthesized with two phosphorothioate linkages on both ends of the arms to increase stability of DNAzymes in cells [26]. A control DNAzyme was designed by inverting catalytic core sequence. In addition, a fluorescein isothiocyanate (FITC)-labeled DNAzyme was used for cellular uptake study [27]. 

**Figure 1:**
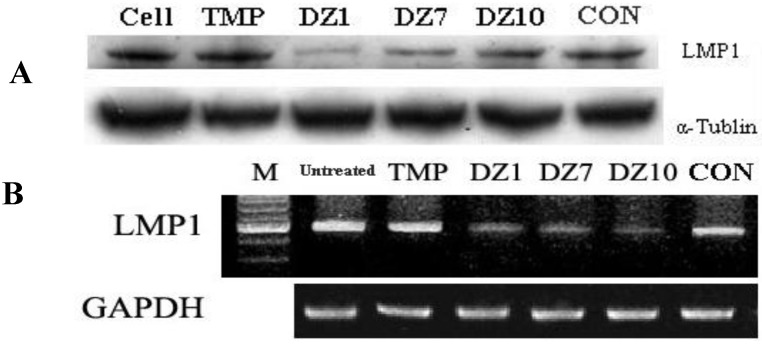
A: Inhibition of LMP1 expression in CNE1-LMP1 cells by DNAzymes. CNE1-LMP1 cells were transfected with DNAzymes or control ODN, total cell lysates and RNA were analyzed by Western blot (A) and RT-PCR (B).

### 2.2. Down-regulation of LMP1 Expression in NPC Cells

The DNAzyme delivery has been the major hurdle in assay of its efficacy. Many types of delivery agents have been widely used for this purpose, including liposome, nanoparticles or electroporation [28]. In our study, to facilitate delivery of DNAzyme oligonucleotides into cells, a cationic porphyrin, TMP, was used as a transfection reagent for intracellular delivery [29], and TMP was shown to be a simple and efficient transfection reagent due to its high complexing efficiency, low cellular toxicity and preferential nuclear uptake [27,30]. 

By targeting the LMP1 mRNA, 13 DNAzymes were transfected into LMP1 positive cells, the data showed that DNAzymes DZ1, DZ7 and DZ10 strongly inhibited LMP1 protein expression compared with the controls, and confirmed that DNAzymes could inhibit LMP1 expression in a dose-dependent manner. In other NPC cell line CNE1-LMP1, three active DNAzymes also inhibited the LMP1 protein expression and reduced the LMP1 mRNA level (Figure 1) [27,30].

### 2.3. Impact of Dz1 on Major Signal Pathways in NPC Cells

**Figure 2:**
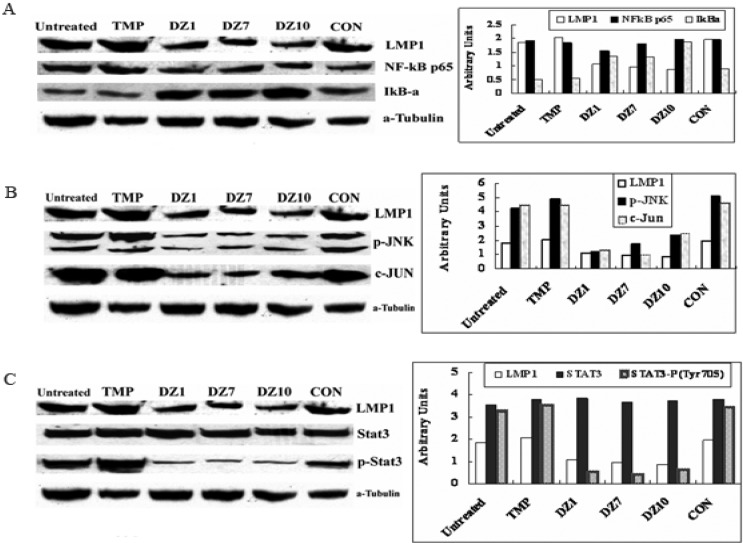
Effect of DNAzymes on NF-κB, AP-1 and STAT3 signal transduction pathways in CNE1-LMP1 cells. Cells were transfected with DNAzymes or Control ODN for 24 h, total cell lysates were analyzed by Western blot respectively (left panel) and the protein expression level was quantitated by densitometry (right panel). (A) NF-κB pathway; (B) c-jun/JNK pathway; (C) Stat3 pathway.

To investigate the effect of down-regulation of LMP1 expression on the down-stream pathways, we analyzed three key signal pathways which are abnormally activated by LMP1 in NPC Cells. LMP1-mediated activation of NF-κB is one of the major signal transduction pathways involved in cell growth and apoptosis in the EBV-associated NPC [31,37,38]. As shown in Figure 2, suppression of LMP1 expression by DNAzymes could reduce the degradation of Ik-Bα, resulting in the loss of NF-κB activity. 

At the same time, DNAzymes could strongly inhibit the AP1 signal transduction pathway via suppressed p-JNK and c-Jun expression. When the JAK/STAT signal pathway was further investigated, it was found that DNAzyme-mediated suppression of LMP1 could markedly decrease the level of phosphorylated Stat3, while total Stat3 level remained unchanged. Taken together, the targeting LMP1 DNAzymes could act upon the cells through three major signal transduction pathways abnormally activated by LMP1 in NPC cells [30]. In our recent studies, the biological activity of the DZ1 was further demonstrated to be mediated through blocking the AKT and EGFR signal pathways, which may be associated with the proliferation and apoptosis of NPC cells.

Oncoprotein 18/stathmin (Op18/stathmin) plays a crucial role in maintaining cell function by regulating microtubule dynamics, especially for the entry into mitosis. Phosphorylated Op18/stathmin promotes microtubule polymerization to form the mitotic spindle, which is essential for chromosome segregation and cell division [32,33]. We showed that inhibition of LMP1 expression by DZ1 attenuated cdc2 kinase activity and the interaction of cdc2 with Op18/stathmin in CNE1-LMP1 cells, and promoted microtubule depolymerization. This finding not only added novel aspects of the LMP1 regulation network but also elucidates the molecular mechanisms of LMP1 that leads to carcinogenesis [34]. 

**Figure 3:**
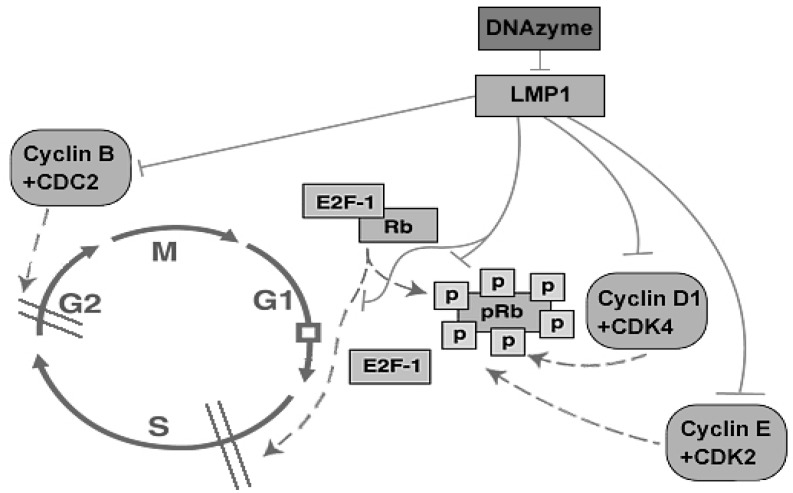
Possible mechanisms for the S phase arrest caused by down-regulation of the LMP1 expression by DZ1 through G1/S and G2/M checkpoint pathways.

We also showed that DZ1 could induce S phase arrest in NPC cells [30]. To investigate the mechanisms of the S-phase arrest, we provided further data showing that DZ1-mediated inhibition of LMP1 expression led to down-regulation cyclin D1 through EGFR and STAT3 signal pathways. In addition, it was observed that expression of CDK4, cyclinE and CDK2 were also affected. Interestingly, the interaction between cyclinD1 and CDK4 as well as the activity of the complex; and Rb protein phosphorylation at Ser780 site were all decreased. E2F1 is an important regulator for entry into S phase [35]. It was shown that LMP1 expression could enhance phosphorylation of p105 Rb to upregulate E2F1 expression and increase E2F1 ability to transactivate the downstream signal molecules through inhibiting p16INK4A expression [36]. Our results showed the DNAzyme could suppress the Rb/E2F1 pathway and markedly down-regulate the expression of E2F1. Thus, downregulation of the expression of LMP1 by DZ1 could decrease the expression of the G1/S related molecules and disrupt their interactions and activities, which consequentially suppressed the pRb-E2F pathway leading to cell cycle arrest through the restriction point in the late G1 phase. Furthermore, the suppression of LMP1 by DZ1 could markedly decrease the cdc2 kinase activity, which led to cell cycle arrest through the restriction point in G2 phase. Taken together, our data suggested that the S phase arrest caused by DZ1 is likely through the G1/S and G2/M checkpoints (Figure 3). 

**Figure 4:**
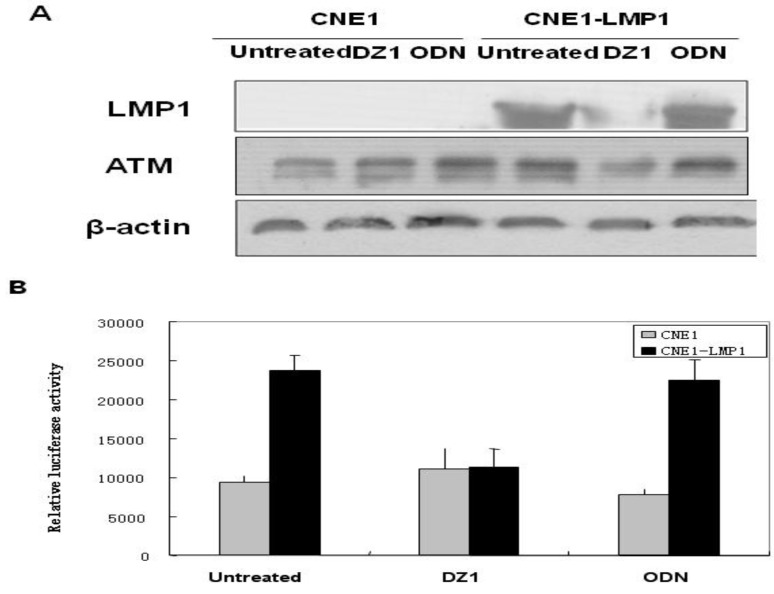
Effect of inhibition of LMP1 expression by DZ1 on the level of ATM. A, CNE1 and CNE1-LMP1 cells were transfected with DNAzyme or control ODN and incubated for 24 h, total cell lysates were analyzed by Western blot. B, Comparison of transcriptional activation of the ATM promoter inNPC cells. The plasmid carrying luciferase reporter gene with the ATM promoter was transiently transfected into CNE1 and CNE1-LMP1 NPC cell lines and the luciferase assay was performed. The data represent the mean±SD of the three independent experiments performed in triplicate.

ATM as a member of a coordinated system that detects DNA breaks; arrests the cells temporarily at G1, S, or G2 checkpoints; and activates DNA repair [39]. Cells lacking functional ATM protein show increased sensitivity to ionizing radiation (IR) and other genotoxic events [40]. Our experiments showed that DZ1 indeed inhibited the ATM expression through the NF-κB pathway by decrease of the binding of NF-κB transcription factor to the ATM promoter (Figure 4), and increased radiosensitivity in LMP1-positive NPC cells. 

### 2.4. Biological Effects of DZ1 on NPC

#### 2.4.1. Proliferation

We experimentally showed that DZ1 inhibited cell proliferation in LMP1 positive NPC cells in MTT assay and the DNAzymes had no effect on the LMP1-negative tumor cell CNE1. When three DNAzymes DZ1, DZ7 and DZ10 were combined and transfected into the cells, an increase in the inhibitory effect was observed compared with any of individual DNAzyme treatment. This combined effect may be due to the multiple targeting of the accessible site within the LMP1 mRNA structures [27,30]. 

To further investigate the cause of the reduced cell proliferation rate in the DNAzymes -treated CNE1-LMP1 cells, flow cytometry was employed to study the effect of the DNAzymes on cell cycle. The results showed that DNAzyme-treated CNE1-LMP1 cells were accumulated in S phase at 24 h post transfection with a significant reduction of cells in G2/M and G0/G1 phases, while no effect was observed on cell cycle of LMP1-negative tumor cell CNE1. These results indicated that the DNAzymes could inhibit cell proliferation in a target-specific manner. To distinguish whether the increase in the proportion of S-phase cells was due to more cells entering S phase or being arrested at S phase, we further conducted BrdU incorporation assay to measure the number of cells entering phase S. The results showed that the number of the BrdU-positive cells was fewer in the DNAzyme-treated cultures than that of control cultures, suggesting that the increase of cells at S phase was caused by cell arrest at S stage of the cell cycle [30].

#### 2.4.2. Apoptosis

**Figure 5:**
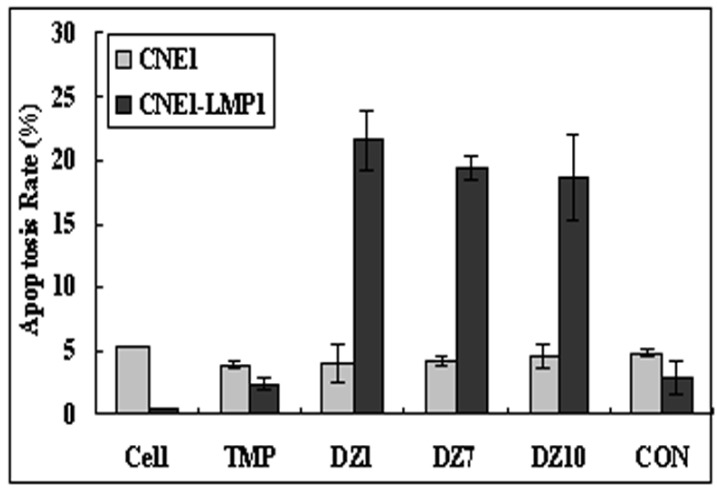
Promotion of apoptosis in LMP1 positive NPC cells by Dz1. Cells were transfected with DNAzymes or controls, The apoptotic rates (sub-G1 population) of CNE1-LMP1 were measured by FACS analyses (values are the means ± SD of three replicates, *p < 0.05 compared with TMP [30].

We further showed that DZ1 promoted apoptosis in LMP1 positive NPC cells (Figure 5). Molecular analyses showed that DNAzymes could concomitantly downregulate the expression of Bcl-2 gene in DZ1 treated cells, and confirmed that DZ1 could induce the release of cytochrome c from mitochondria, which is the hallmark of the apoptosis. The analyses of the activities of Caspase-3, -9 and -8 demonstrated that DZ1 could increase the activity of all three caspaseses with Caspase-3 and Caspase-9 being more significant, suggesting that the DNAzymes induced apoptosis in CNE1-LMP1 cells predominantly via the mitochondria pathway [27,30]. Our recent studies showed that the AKT signal pathway may be associated with the apoptosis induced by DZ1 (unpublished data). 

#### 2.4.3. Metastasis

**Figure 6:**
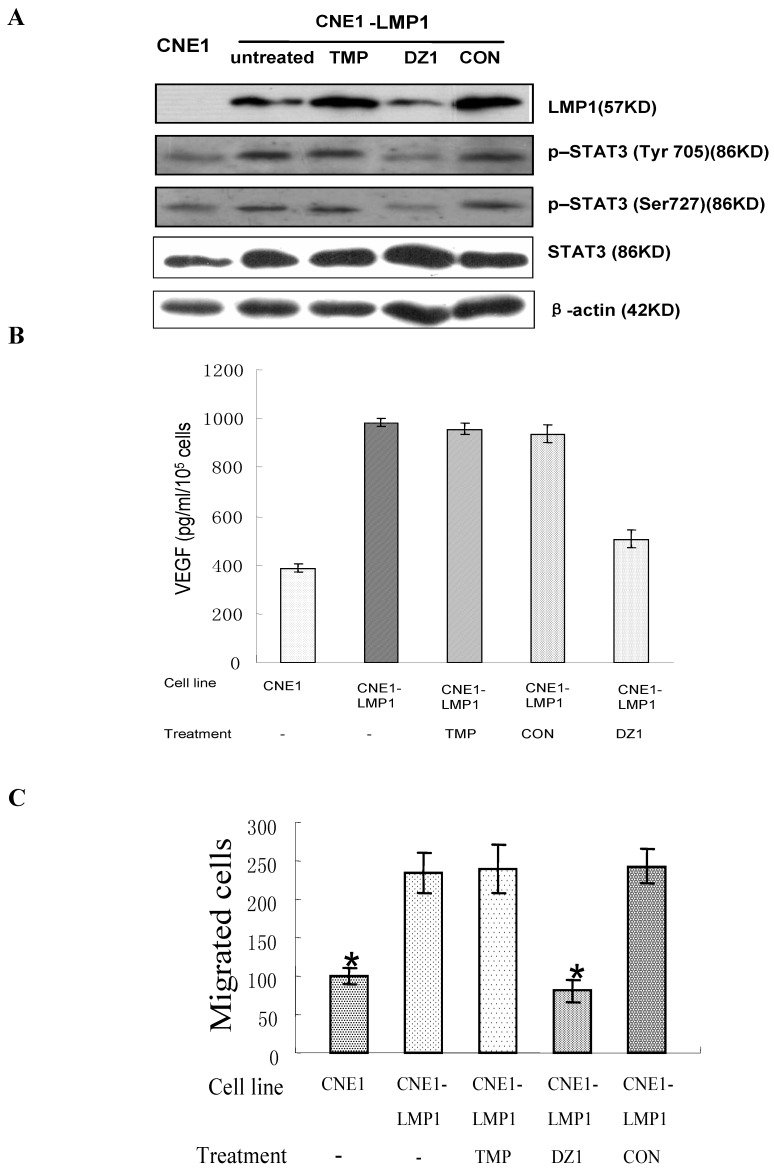
DZ1 suppression of metastasis potential in LMP1 positive NPC cells. A: DZ1 decreased STAT3 phosphorylation. NPC cells were transfected with DNAzyme or controls. After 24 h, LMP1 expression and STAT3 levels was determined by immunoblotting with antibodies specific for LMP1, phosphorylated STAT3 Tyr705 and STAT3 Ser727. B: Soluble VEGF were collected from conditioned medium and quantitatively determined by ELISA. C: Migration assay. The number of cells that had migrated through the pores was quantified by counting five independent visual fields using a 20× microscope objective. Three independent assays were performed. Each point shows the mean ±SD.

STAT3 is a master transcriptional regulator in proliferation and apoptosis and is newly implicated in angiogenesis and invasiveness, which, in turn, are likely to contribute to the highly invasive character of NPC [41]. Our experiments showed that DZ1 could concomitantly decrease Tyr705 and Ser727 phosphorylation of STAT3 in CNE1-LMP1 through JAK3 and extracellular signal-regulated kinase 1/2 (ERK1/2) pathways, but had no effect on STAT3 expression. Furthermore, DZ1 repressed the vascular endothelial growth factor (VEGF) expression and secretion via the JAK/STAT and mitogen-activated protein kinase (MAPK)/ERK signaling pathways (Figure 6). Cell biological evidence showed that DZ1 significantly inhibited the migration ability by 65% in CNE1-LMP1, suppression of LMP1-regulated STAT3 signaling decreased the ability for matrigel transmembrane migration. The increased cell motility and invasion are correlated with increased metastasis potential. Thus, the DNAzyme could inhibit STAT3 activation via JAK3 and ERK1/2, leading to suppression of the NPC potential of migration and invasion.

#### 2.4.4. Anti-tumor effect

**Figure 7:**
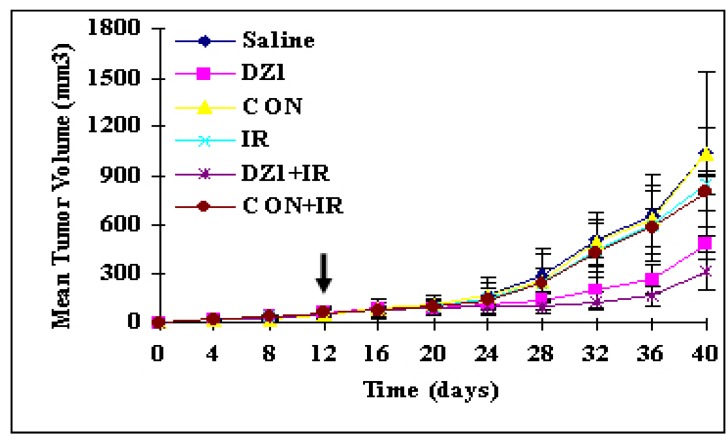
DZ1-mediated inhibition of tumor growth and enhanced radiosensitivity *in vivo*. Six mice per group were injected DZ1 intratumorally twice weekly and 5Gy irradiation treatments were performed.

In order to determine whether Dz1 could influence the growth of NPC *in vivo*, we assessed the effect of Dz1 on pre-established CNE1-LMP1 carcinomas grown in nude mice. The result showed that Dz1 suppressed the tumor growth, whereas control-ODN had no effect (Figure 7). When the target gene expression was analyzed by immunohistochemical staining on the cross-sections of paraffin-embedded formalin-fixed tissues from different treatment groups, it was shown that LMP1 expression in the tumor tissues was significantly reduced. To gain additional evidence of DZ1-mediated effects, Bcl-2 expression was also examined and shown to be markedly reduced in the DNAzyme-treated group in comparison with the control groups [30]. TUNEL assay further demonstrated the presence of apoptosis in the groups treated with DZ1. Together, the DNAzyme could inhibit its target gene LMP1 expression in tumors, which led to a significant suppression of tumor growth of NPC *in vivo* [30].

#### 2.4.5. Radiosensitization

NPC is highly radiosensitive, therefore radiotherapy or radiotherapy combining with chemotherapy are the main treatment strategies of the NPC [42]. We used three parameters to evaluate if there existed a combined effect of DNAzymes and irradiation on CNE1-LMP1cells. In FACS-based assay, it was shown that the combined treatment could significantly increase the apoptotic rate compared to the treatments by DNAzymes or ionized radiation (IR) alone; When MTT and colony-forming assays were performed, both the cell proliferation and colony-forming ability were inhibited more significantly in the combinational treatment than in either DNAzyme or IR treatment alone, confirming that down-regulation of LMP1 could sensitize the CNE1-LMP1 cells to irradiation.

**Figure 8:**
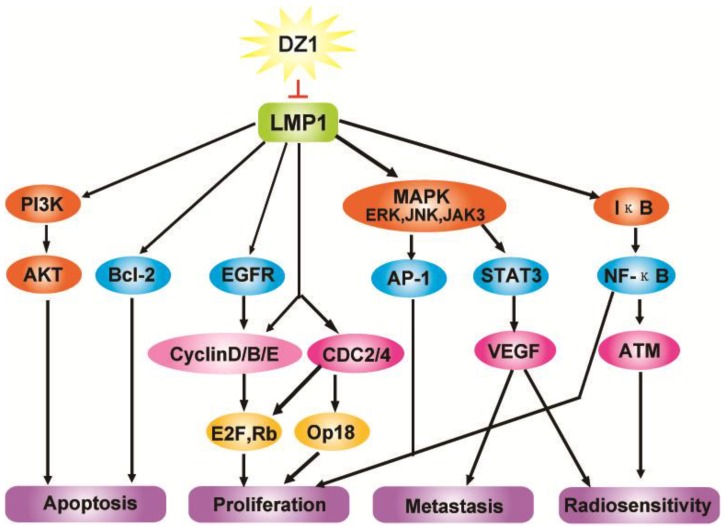
Impact of Dz1 on signal pathways and associated cell functions in NPC cells.

Furthermore, *in vivo* experiments demonstrated the efficient uptake of FITC-labeled DNAzymes by tumor cells. When a suboptimal dose of radiation treatment was combined with the DNAzyme injections, the further tumor reduction was observed in the DZ1 + IR group , while the radiation treatment showed only a minor effect on the tumor growth(Figure 8) , these result showed that the DZ1 could enhance radiosensitivity *in vivo* [30]. 

## 3. Conclusions

The majority of oncogenic signaling pathways converge on sets of transcription factors that ultimately control gene expression patterns resulting in tumor formation and progression as well as metastasis. LMP1 is one such a transcriptional factor derived from EBV that functions as a central point in the signal transduction network in NPC (Figure 8). Therefore, it represents a highly desirable and logical point of therapeutic interference in NPC development and progression. In our studies, the use of DNAzymes not only facilitated the validation of the LMP1 as a potential drug target, but also provided solid data to support a further development of the LMP1-targeted DNAzyme in a clinical setting.
